# Strontium-substituted sub-micron bioactive glasses inhibit ostoclastogenesis through suppression of RANKL-induced signaling pathway

**DOI:** 10.1093/rb/rbaa004

**Published:** 2020-03-30

**Authors:** Deqiu Huang, Fujian Zhao, Wendong Gao, Xiaofeng Chen, Zhouyi Guo, Wen Zhang

**Affiliations:** r1 MOE Key Laboratory of Laser Life Science & SATCM Third Grade Laboratory of Chinese Medicine and Photonics Technology, College of Biophotonics, South China Normal University, Guangzhou 510631, Guangdong, China; r2 Department of Biomedical Engineering, School of Materials Science and Engineering, South China University of Technology, Guangzhou 510641, Guangdong, China; r3 Department of Medical Biotechnology, School of Basic Medical Sciences, Guangzhou University of Chinese Medicine, Guangzhou, Guangdong, China

**Keywords:** strontium-substituted sub-micron bioactive glass, osteoclastogenesis, RANKL signaling pathway, RAW264.7

## Abstract

Strontium-substituted bioactive glass (Sr-BG) has shown superior performance in bone regeneration. Sr-BG-induced osteogenesis has been extensively studied; however, Sr-BG-mediated osteoclastogenesis and the underlying molecular mechanism remain unclear. It is recognized that the balance of osteogenesis and osteoclastogenesis is closely related to bone repair, and the receptor activators of nuclear factor kappaB ligand (RANKL) signaling pathway plays a key role of in the regulation of osteoclastogenesis. Herein, we studied the potential impact and underling mechanism of strontium-substituted sub-micron bioactive glass (Sr-SBG) on RANKL-induced osteoclast activation and differentiation *in vitro*. As expected, Sr-SBG inhibited RANKL-mediated osteoclastogenesis significantly with the experimental performance of decreased mature osteoclasts formation and downregulation of osteoclastogenesis-related gene expression. Furthermore, it was found that Sr-SBG might suppress osteoclastogenesis by the combined effect of strontium and silicon released through inhibition of RANKL-induced activation of p38 and NF-κB pathway. These results elaborated the effect of Sr-SBG-based materials on osteoclastogenesis through RANKL-induced downstream pathway and might represent a significant guidance for designing better bone repair materials.

## Introduction

Multifunctional bioactive materials including calcium phosphate, bioceramics and bioactive glasses (BGs) have attracted much attention for bone defects repair owing to their properties of bone conductivity and inductivity [1–4]. Among these materials, BGs have the advantage that they can not only strongly bond to bone through surface formation of carbonated apatite (HCA) layer [5], but can also release bioactive ions such as silicon (Si), calcium (Ca), phosphate (P) to promote bone regeneration during the degradation of materials [6–9]. Since the first discovery of 45S5 BGs by Hench, they have been used in clinical bone repair for many years [10]. However, the products used in clinic are mainly prepared by melting method, which has dense structure and is not conducive to ion dissolution and bone tissue growth [11]. In our previous work, micro–nano bioactive glass (MNBG) with good monodispersity and controlled morphology was synthesized using sol–gel technique combined with template self-assembly method. The MNBG shows higher specific surface area and superior biological activity compared to conventional BGs [12, 13]. In order to further improve the bone repair function of MNBG, strontium-incorporated MNBG (Sr-MNBG) was developed which allows the sustained release of strontium from MNBG [14, 15]. It was found that enhanced bone regeneration has been achieved with the application of strontium-substituted sub-micron bioactive glass (Sr-SBG) both *in vitro* and *in vivo* [15, 16]. Moreover, Sr-SBG has bidirectional function in the regulation of bone regeneration including increased anabolic activity of osteoblasts as well as declined catabolic activity of osteoclasts [17, 18]. At present, Sr-SBG-induced osteogenesis has been extensively studied [17, 19, 20], however, Sr-SBG-mediated osteoclastogenesis and the underlying molecular mechanism remains unclear. It is recognized that osteogenesis and osteoclastogenesis are two key processes which are closely related to bone repair [21–23]. In bone repair processes following implantation of materials *in vivo*, the bone resorption rate will be greater than the bone formation rate if the osteoclast is overactive, which is detrimental to bone repair. Therefore, to evaluate the bone regeneration effect of bone repair materials, it is not only necessary to study their interaction with osteogenic cells, but also essential to investigate the function of materials on osteoclast differentiation and its molecular mechanism. Hence, the impact of Sr-SBG on osteoclast differentiation and the precise molecular mechanism need to be studied.

Bone remodeling is regulated by osteogenesis and osteoclastogenesis through continuous osteoblastic formation of new bone and osteoclastic resorption of old bone [24, 25]. Disruption of the balance between osteogenesis and osteoclastogenesis can lead to skeletal malformations, such as osteoporosis and sclerosis [26]. Receptor activators of nuclear factor kappaB ligand (RANKL) have been demonstrated to play a key role in the regulation of osteoclastogenesis. When membrane RANKL or soluble RANKL is bound to the RANK receptor on the surface of osteoclast precursor cells, a series of downstream signaling pathways will be activated, such as the NF-κB, p38, JNK, ERK and Src pathway. Subsequently, the expression of osteoclastogenesis specific genes is up-regulated which further promotes osteoclasts differentiation and activation through the regulation of transcription factor c-Fos, NFATc1 and myc [27–30].

Sr-SBG extract mainly contains four bioactive ions including Ca, Si, P and Sr. It has been reported that the Ca^2+^ and Sr^2+^ ion may affect the osteoclast differentiation through Calcinemin-NFATc signaling pathway [31, 32]. The increasement of the intracellular Ca^2+^ ion can activate calcineurin and then induce dephosphorylation of NFATc1 present in the cytoplasm. The dephosphorylated NFATc1 can translocate to nucleus to induce the transcription of the osteoclastogenesis-related genes. It has been found that the bioactive ceramic material containing Si ions could inhibit osteoclast differentiation depending on the concentration of Si, while the specific molecular mechanism is still unknown [33]. In addition, the role of Sr ion in inhibiting the differentiation of osteoclasts is thought to be related to the blocking of the NF-κB signaling pathway [34]. The effect of P ions on osteoclast differentiation has not been reported yet. Since these elements may all play important roles in osteoclastogenesis, it is reasonable to believe that Sr-SBG might inhibit osteoclastogenesis through multiple signaling pathways.

Herein, we synthesized Sr-SBG with 6% Sr molar content and SBG microspheres. And we set up three experimental groups including Sr-SBG dissolution extracts group, SBG dissolution extract group, as well as SrCl_2_ group which has identical amount of Sr ion concentration with Sr-SBG dissolution extract group. The effects of materials from three groups on RANKL-mediated osteoclastogenesis from RAW 264.7 cells *in vitro* were investigated and compared. We also examined the precise molecular mechanism of the role of Sr-SBG extract in osteoclast differentiation *in vitro* by examining the RANKL-induced multiple downstream signaling pathways involved in this process. The findings of this work might show synergistic effect of BGs dissolution and strontium on osteoclastogenesis, and offer possible mechanistic explanation for the effect of Sr-SBG on enhanced bone regeneration. Furthermore, it might provide a significant guidance for designing better bone repair materials.

## Materials and methods

### Materials

Tetraethyl orthosilicate (TEOS), calcium nitrate tetrahydrate (CN), absolute ethanol and strontium nitrate (SN) were supplied by Guangzhou Chemical Reagent Factory, China. Triethylphophate (TEP), strontium chloride (SrCl_2_) and dodecylamine (DA) were purchased from Shanghai Aladdin Co. Ltd., China. All of these reagents are analytical grade and were used directly without other treatment.

### Synthesis and characterization of SBG and Sr-SBG

SBG and Sr-SBG were synthesized as previously described by the combined template self-assembly method and sol–gel technique with DA served as template agent and catalyst [13, 14, 35]. SBG was composed of SiO_2_, CaO and P_2_O_5_ with the molar percentage of 60, 36 and 4, respectively. And Sr-SBG was composed of SiO_2_, CaO, SrO and P_2_O_5_, with the molar percentage of 60, 30, 6 and 4, respectively. Briefly, a mixture of 80 ml ethanol and 25 ml distilled water was stirred for 10 min at a constant temperature of 40°C. Afterward, 4 g DA was added to the above mixture with continuous stirring for 10 min. Then, 8 ml TEOS was dropwise added to the absolutely dissolved solution under vigorous stirring. After an interval of 30 min, another 8 ml TEOS was added. After the complete addition of TEOS for 30 min, calculated amount of TEP, CN and SN (only for Sr-SBG) was dropwise added to the solution successively with 30 min intervals by vigorous stirring. The resulting milky mixture was aged for 6 h after being stirred vigorously for another 3 h. Finally, the suspension was centrifuged at 4000 rpm for 5 min and the white precipitate was collected. The white precipitate was washed three times each with absolute ethanol and distilled water. Then the precipitate was freeze-dried for 48 h. Subsequently, the freeze-dried products were sintered at 650°C for 3 h in air and the SBG and Sr-SBG micron particle powders were obtained after grinding. The materials extract of SBG and Sr-SBG was prepared as previously described. Typically, 10 mg SBG and Sr-SBG micron particles were sterilized and mixed with 10 ml Dulbecco modified Eagle medium (DMEM). The mixture was shaken at 120 rpm for 48 h with a constant temperature of 37°C. The SBG and Sr-SBG extracts were collected by filtration with 0.22 μm syringe filter after centrifugation. A percent of 10% (v/v) fetal bovine serum (FBS) and 1% (v/v) penicillin/streptomycin were added to SBG and Sr-SBG extracts for cell culture in the subsequent procedures. Morphology and energy-dispersive X-ray spectroscopy (EDX) characterization of SBG and Sr-SBG were performed on a scanning electron microscopy (SEM) equipped with an EDX spectrometer (DSM 982-Gemini, Zeiss, Germany). Zeta potential and particle size distribution were conducted on Zetasizer Nano ZS (Malvern Instruments, UK). Element composition of SBG and Sr-SBG were tested by X-ray photoelectron spectroscopy (Kratos Axis UlraDLD, UK). The ion concentrations of SBG and Sr-SBG extracts were examined by inductively coupled plasma-atomic emission spectrometry (ICP-AES) (PS1000-AT, Leeman, USA).

### Cell culture

RAW 264.7 cells were purchased from American Type Culture Collection (ATCC). DMEM containing 10% FBS and 1% (v/v) penicillin/streptomycin was used for the culture of RAW 264.7 cells. RAW 264.7 cells were cultured in an 5 CO_2_ incubator at 37°C and passaged by gently scraping when the cells reached a approximately confluence of 85%. Cells from the third to seven passages were applied for osteoclast differentiation experiment. Three experimental groups including SBG extract group, Sr-SBG extract group and SrCl_2_ group with the same Sr concentration with Sr-SBG extract as characterized by ICP-AES were established in the following experiments.

### Cell proliferation

To evaluate the proliferation of RAW 264.7 cells affected by SBG, Sr-SBG extracts and SrCl_2_ with identical Sr concentration, RAW 264.7 cells were seeded at a density of 3 × 10^3^ cells/well in 96 well plate. After 24 h of attachment, cells medium was replaced by different mediums from three experimental groups. After another 1, 3 and 5 days, cell was harvested for proliferation assay by cell counting kit-8 assay (CCK-8, Dojindo Laboratories, Japan) (*N* = 5). The optical density (OD) values at 450 nm of each well were measured by Microplate Reader (Thermo 3001, Thermo Scientific, USA).

### Tartrate-resistant acid phosphatase staining and TRAP activity quantification assay

RAW 264.7 cells with 3000 cells/well were seeded in 96-well plates. After 24 h of attachment, cells were incubated with SBG extract, Sr-SBG extract or SrCl_2_ containing 50 ng/ml RANKL (R&D) for 5 days. Cell culture medium was refreshed every 2 days. Then, cells were rinsed twice with phosphate buffer saline (PBS), and fixed with 4% paraformaldehyde for tartrate-resistant acid phosphatase (TRAP) staining using a commercial TRAP kit (387-A, Sigma-Aldrich) under the instruction of the manufacturer’s protocol. For TRAP activity quantification assay, cells were fixed with 4% formalin and 95% ethanol for 15 and 3 min, respectively. After that, working solution containing 10 mM sodium tartrate and p-nitrophenylphosphate in 10 mM citrate buffer (pH 4.6) were added and incubated for 1 h. Finally, stop solution (5 M NaOH) was added and the OD value at 405 nm was recorded.

### Observation of F-actin ring

RAW 264.7 cells with 3000 cells/well were seeded in 96-well plates. After 24 h of attachment, cells were incubated with SBG extract, Sr-SBG extract or SrCl_2_ containing 50 ng/ml RANKL (R&D) for 5 days. Cell culture medium were refreshed every 2 days. Then, cells were rinsed twice with PBS, and fixed with 4% paraformaldehyde for 20 min. Subsequently, 0.1% Triton-PBS was used for cell permeabilization for 15 min. After blocking with 10% FBS–PBS, cells were incubated with fluorescein isothiocyanate (FITC)-labeled phalloidin (Sigma-Aldrich) for 1 h. Cell nuclei were stained with 4,6-diamidino-2-phenylindole (DAPI) for 5 min. Eventually, F-actin rings were photographed by fluorescence microscope (Eclipsc Ti–U, Nikon, Japan).

### Osteoclastogenesis-related immunofluorescence analysis

RAW 264.7 cells with 3000 cells/well were seeded in 96-well plates and attached for 24 h. Then, cell medium was changed by SBG extract, Sr-SBG extract or SrCl_2_ containing 50 ng/ml RANKL for 24 h. Cells were harvested and rinsed with PBS twice. After being fixed with 4% paraformaldehyde and permeabilized with 0.1% Triton-PBS, cells were blocked in 10% FBS for 1 h and were then incubated with anti-NFATc1 primary antibody (1:100 dilution; Santa Cruz Biotechnology) at 4°C overnight. After three times of rinsing with PBS, cells were further incubated with Cy3-conjugated donkey anti-goat immunoglobulin G (1:200 dilution; CWBIO, China). Then DAPI was used to stain the cell nuclei. The immunofluorescence results were observed under fluorescence microscope.

### Osteoclastogenesis-related gene expression

RAW 264.7 cells with 1 × 10^5^ cells/well were seeded in 24-well plates and attached for 24 h. Afterward, cell medium was changed by SBG extract, Sr-SBG extract or SrCl_2_ containing 50 ng/ml RANKL for 24 h and 72 h. Then, RAW 264.7 cells were collected for total RNA isolation. Total RNA was extracted from the treated RAW 264.7 cells using a commercial HiPure Total RNA Micro Kit (Magen) according to the instructions of the manufacturer. Reverse transcription was performed using the Takara reagents kit to reverse transcribe total RNA into cDNA. The expression levels of osteoclastogenesis-related gene including NFATc1, c-Fos, TRAP, cathepsin K and MMP-9 were detected by real-time quantitative polymerase chain reaction (RT-qPCR). The primers of osteoclastogenesis-related genes used in this study were listed in Table 1. RT-qPCR was conducted on Quantstudio 6 Flex (Life technologies) with Maxima SYBR Green/ROX qPCR kit (Thermo Scientific). All of the gene expression results were normalized to housekeeping gene glyceraldehyde-3-phosphate dehydrogenase (GAPDH) and were analyzed by the 2^−ΔΔCt^ method.

**Table 1:** Primer sequences of osteogclastogenesis-related genes used in RT-qPCR

Genes	Primer sequences
GAPDH	Forward : 5′-CTCCCACTCTTCCACCTTCG-3′
	Reverse: 5′-TTGCTGTAGCCGTATTCATT-3′
TRAP	Forward: 5′-TACCTGTGTGGACATGACC-3′
	Reverse: 5′-CAGATCCATAGTGAAACCGC-3′
NFATc1	Forward: 5′-GGTAACTCTGTCTTTCTA ACCTTAAGCTC-3′
	Reverse: 5′-GTGATGACCCCAGCATGCA CCAGTCACAG-3′
c-Fos	Forward: 5′-CCAAGCGGAGACAGATCAACTT-3′
	Reverse: 5′-TCCAGTTTTTCCTTCTCTTT CAGCAGAT-3′
Cathepsin K	Forward: 5′-CAGCAGAACGGAGGCATTGA-3′
	Reverse: 5′-CCTTTGCCGTGGCGTTATAC-3′
MMP-9	Forward: 5′-TCCAGTACCAAGACAAAGCCTA-3′
	Reverse: 5′-TTGCACTGCACGGTTGAA-3′

### Protein extraction and Western blot analysis

RAW 264.7 cells were incubated with SBG extract, Sr-SBG extract or SrCl_2_ in the presence of 50 ng/ml RANKL for 7 h. For protein extraction, cells were collected and washed with pre-cold PBS. Then, RIPA lysis (Beyotime) containing Phenylmethanesulfonyl fluoride (PMSF), protease and phosphatase inhibitor cocktail was used to extract the total cellular proteins. After centrifugation at 14 000 g for 30 min, the supernatant was collected for western blot analysis immediately or stored in −80°C. Protein concentrations were measured by a bicinchoninic acid (BCA) protein assay kit (Thermo Scientific). Western blot assay was operated according to previous protocols [15]. Primary antibodies including anti-p38 antibody (Cell Signaling Technology, 1:500), anti-phospho-p-38 antibody (Cell Signaling Technology, 1:500), anti-ERK1/2 (Abcam, 1:1000) antibody, anti-phospho-ERK1/2 antibody (Santa Cruz Biotechnology, 1:500), anti-JNK antibody (proteintech, 1:6000), anti-phospho-JNK antibody (Bioss, 1:1000), anti-IκBα antibody (Cell Signaling Technology, 1:1000) and anti-GAPDH antibody (Cell Signaling Technology, 1:1000) were used in this study. The secondary antibodies were obtained from Abcam with a 1:10 000 dilution. Proteins were electrophoresed through 10% sodium salt-polyacrylamide gel electrophoresis (SDS-PAGE) gels and were then transferred to a polyvinylidene fluoride (PVDF) membrane. After being blocked with 5% skim milk, PVDF membrane was incubated with primary antibodies overnight at 4°C. Then, horseradish peroxidase (HRP)-conjugated secondary antibodies were incubated with membrane for 1 h. Ultimately, the target protein bands were exposed with an electrochemiluminescence (ECL) kit (Beyotime) on Biorad ChemiDoc Touch. Quantitative analysis of the densitometric of the images was performed on AlphaEase FC software with GAPDH used as reference protein.

### Statistical analysis

All of the data were expressed as mean ± standard deviation (SD). The experiments were all repeated for three times. Statistical analysis was carried out using SPSS Statistics version 22 (IBM, USA). The significant differences between groups were analyzed by one-way analysis of variance with least significance difference (LSD) post-test. *P* < 0.05 was regarded as statistically significant.

## Results and discussion

### Characterization of Sr-SBG and SBG and materials extract

**Figure 1:**
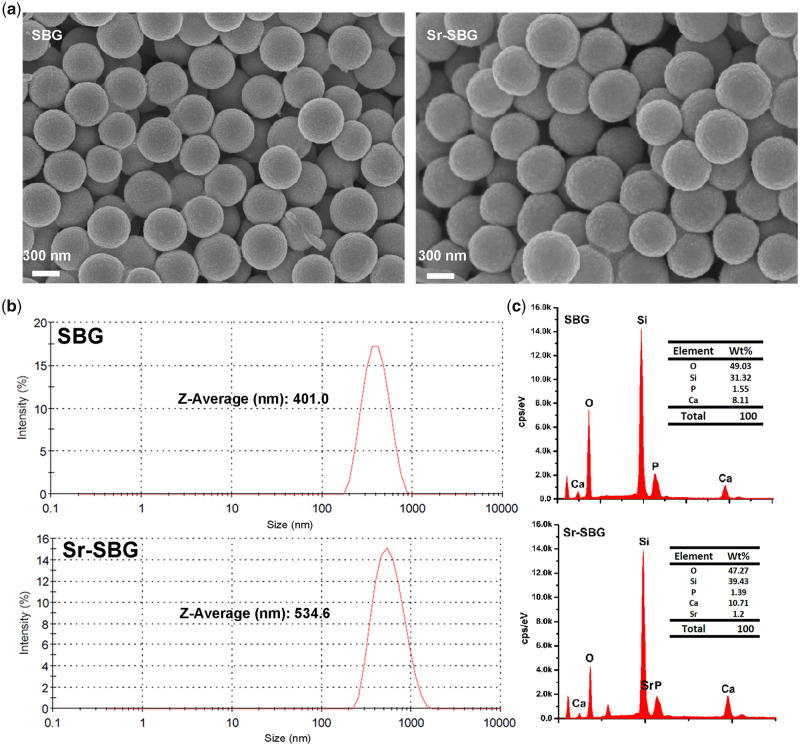
(**a**) SEM images of SBG and Sr-SBG; (**b**) DLS characterization of particle sizes of SBG and Sr-SBG; (**c**) EDX analysis of SBG and Sr-SBG, the inserted tables are element compositions of SBG and Sr-SBG, respectively.

Sub-micron SBG and Sr-SBG particles were successfully prepared by the DA alkali-catalyzed sol–gel method. The surface morphology of sub-micron spheres characterized by SEM was shown in Fig. 1a. It could be observed that SBG and Sr-SBG particles had a uniform structure and good monodispersity. Size distribution and zeta potential of SBG and Sr-SBG detected by dynamic light scattering (DLS) suggested that SBG and Sr-SBG had an average size of 401.0 nm and 534.6 nm, respectively. And zeta potential of SBG and Sr-SBG was 21.3 mV and 29.4 mV, respectively (Fig. 1b and Supplementary Table S1). The particle size of Sr-SBG was obviously increased compared with SBG. The larger particle size of Sr-SBG might be ascribed to much looser network of Sr-SBG than SBG structure, resulting from larger Sr ionic radius than Ca. The element composition determined by EDS (Fig. 1c) and XPS (Supplementary Fig. S1) demonstrated that successful incorporation of Sr element into SBG network. ICP-AES result of material extracts of SBG and Sr-SBG was shown in Table 2. It revealed that Si, Ca and P ion concentration of SBG and Sr-SBG were similar. On the contrary, Si, Ca and P ion concentration of BGs groups and DMEM control group showed distinct differences, with a declined Ca and P ion concentration of BG groups compared with control group. In addition, the Sr ion concentration of Sr-SBG group and SrCl_2_ group was ∼5.518 mg/l.

**Table 2:** Si, Ca, P and Sr Concentration of SBG extract, Sr-SBG extract and SrCl_2_

Groups	Si (mg/l)	Ca (mg/l)	P (mg/l)	Sr (mg/l)
DMEM	0.02	51.85	27.93	0
SBG	31.04	44.29	12.32	0
Sr-SBG	32.35	41.02	10.43	5.518
SrCl_2_	0.02	51.76	27.61	5.525

### Proliferation of RAW 264.7 cells

To evaluate the proliferation of RAW 264.7 cells stimulated by different material groups, RAW 264.7 cells were cultured with materials extract for 1, 3 and 5 days. After being harvested, CCK-8 assay was used to determine the cell proliferation. As can be seen from Supplementary Fig. S2, RAW 264.7 cell proliferation on Days 1 and 3 displayed no obvious difference among the three experimental groups. However, on Day 5, an enhanced proliferation ability of three experimental groups than control group could be observed (*P* < 0.05). There was no distinct difference among the three experimental groups (*P* > 0.05). These results demonstrated that all the materials showed no cytotoxicity to RAW 264.7 cells and had a promotion effect on RAW 264.7 cell proliferation.

### TRAP activity of RANKL-induced RAW 264.7 cells

**Figure 2:**
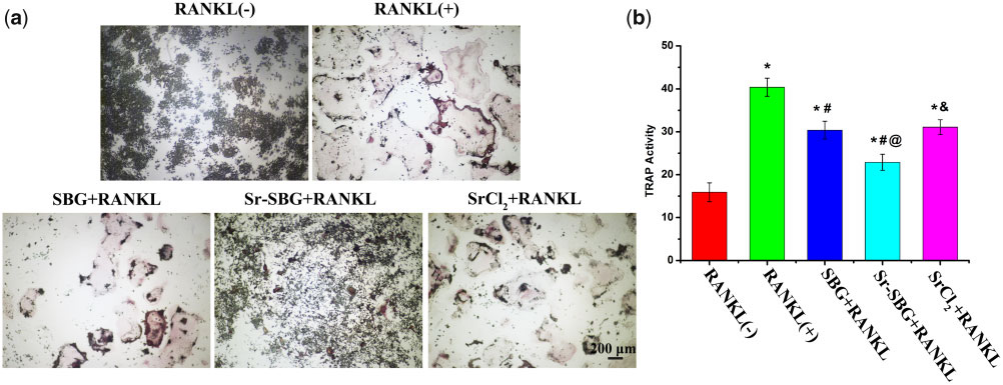
(**a**) TRAP staining of RAW264.7 cells cultured with SBG extract, Sr-SBG extract and SrCl_2_ supplemented with 50 ng/ml RANKL for 5 days; (**b**) TRAP activity quantitative analysis of RAW264.7 cells cultured with SBG extract, Sr-SBG extract and SrCl_2_ supplemented with 50 ng/ml RANKL for 5 days. (*P < 0.05 vs RANKL (−) group; ^#^*P* < 0.05 vs RANKL (+) group; ^@^*P* < 0.05 vs SBG+RANKL group; ^&^*P* < 0.05 vs Sr-SBG+RANKL group).

TRAP activity is one of the criteria to evaluate the effect of materials on osteoclast differentiation. Figure 2a exhibited the TRAP staining of SBG extract, Sr-SBG extract and SrCl_2_ medium co-cultured with 50 ng/ml RANKL-induced RAW264.7 cells for 5 days. It could be seen that no TRAP-positive osteoclast cells were formed without supplement of RANKL, while the formation of giant TRAP-positive osteoclast cells were displayed after the treatment of RANKL for 5 days. All of three experimental groups including SBG extract, Sr-SBG extract and SrCl_2_ could inhibit the formation of osteoclast induced by RANKL, with the findings of decreased osteoclast number and volume than RANKL (+) group. The inhibitory effect of Sr-SBG group on osteoclast differentiation was the strongest, with the phenomenon that large osteoclasts were rarely seen in Sr-SBG group. The quantitative results of TRAP activity were shown in Fig. 2b. The results of TRAP activity were consistent with the results of TRAP staining. The TRAP activity in Sr-SBG group, SBG group and SrCl_2_ group was significantly declined than that in RANKL-induced positive group, with Sr-SBG group exhibited much lower TRAP activity than SBG group and SrCl_2_ group. According to the results of TRAP staining and TRAP quantitative analysis, it could be concluded that Sr-SBG had the strongest inhibitory effect on osteoclast differentiation, and SBG and SrCl_2_ also had inhibitory effect on osteoclast differentiation to some extent, while there was no significant difference between these two groups.

### Inhibition of Sr-SBG on osteoclastic F-actin ring formation mediated by RANKL

**Figure 3:**
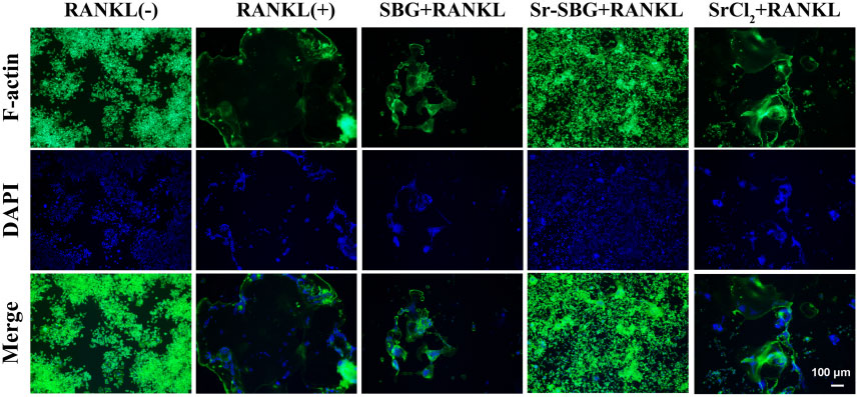
F-actin staining of RAW264.7 cells cultured with SBG extract, Sr-SBG extract and SrCl2 supplemented with 50 ng/ml RANKL for 5 days.

F-actin staining was used to observe the actin fiber rings of osteoclasts. The results in Fig. 3. showed that in the RANKL (−) group without RANKL induction, RAW264.7 still displayed a small round cell. In contrast, in the RANKL (+) group with the induction of RANKL for 5 days, macrophages gradually merged into dozens of nuclear giant cells with the formation of large actin rings. However, under the stimulation of Sr-SBG, SBG and SrCl_2_, the osteoclast differentiation induced by RANKL was affected, resulted in smaller volume of F-actin ring formation. Moreover, the inhibitory effect of Sr-SBG on macrophage osteoclast differentiation was the most obvious, with little actin ring formation compared with other two groups. The inhibitory effect between SBG and SrCl_2_ on F-actin ring formation induced by RANKL showed no statistical differences difference (*P* > 0.05).

### Inhibitory effect of Sr-SBG on RANKL-induced osteoclastogenesis-related gene expression

**Figure 4:**
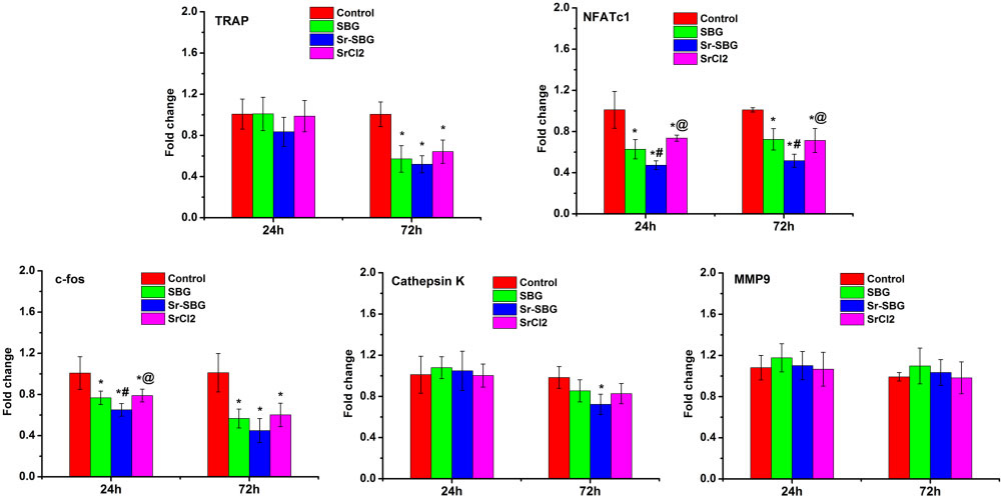
Osteoclastogenesis related genes expression of RAW264.7 cells cultured with SBG extract, Sr-SBG extract and SrCl_2_ supplemented with 50 ng/ml RANKL for 24 and 72 h. (*P < 0.05 vs control group; ^#^*P* < 0.05 vs SBG group; ^#^*P* < 0.05 vs Sr-SBG group).

Osteoclastogenesis-related gene expression induced by RANKL is closed related to osteoclast differentiation. The mRNA expression of TRAP, NFATc1, c-Fos, Cathepsin K and MMP-9 were examined by RT-qPCR. The results are shown in Fig. 4. It could be found that the expression of NFATc1 and c-Fos in Sr-SBG group, SBG group and SrCl_2_ group was significantly lower than that in RANKL (+) control group at 24 h. At 72 h, the expression of TRAP, NFATc1, c-Fos and Cathepsin K was significantly downregulated in Sr-SBG group, SBG group and SrCl_2_ group. Moreover, the results showed that Sr-SBG could inhibit the gene expression level of NFATc1 and c-Fos more strongly than SBG and SrCl2 (*P* < 0.05).

### The inhibition of Sr-SBG on RANKL-induced signaling pathways in RAW 264.7 cells

**Figure 5:**
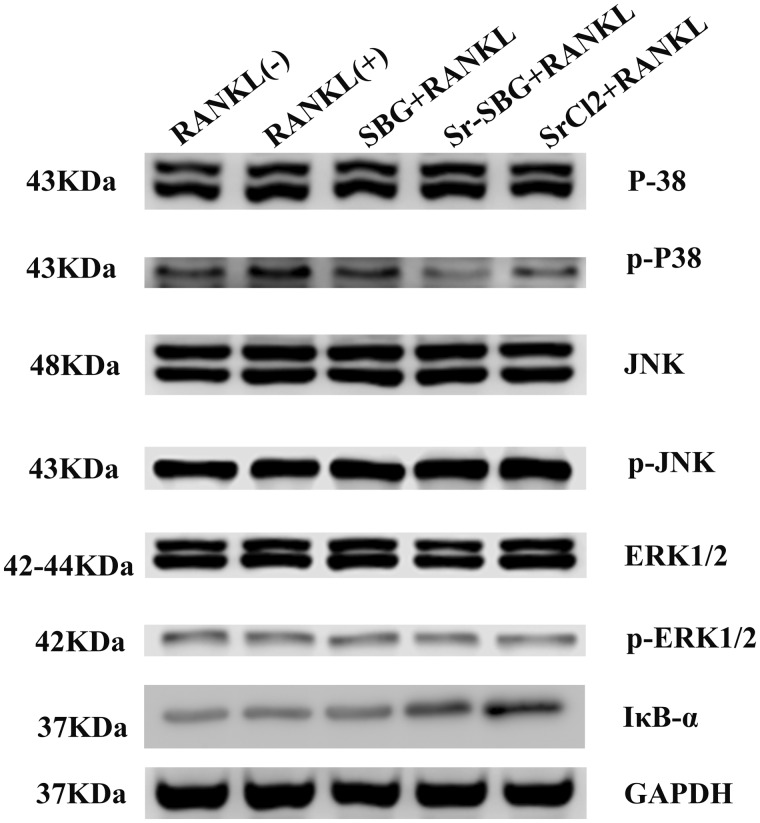
Protein expression of MAPK signaling pathway of p38, JNK, ERK1/2 and their phospholated protein, and IκB-α by Western blot assay in RAW264.7 cells cultured with SBG extract, Sr-SBG extract and SrCl_2_ supplemented with 50 ng/ml RANKL for 7 h.

The molecular mechanism of Sr-SBG inhibiting osteoclastic differentiation of RAW264.7 cells induced by RANKL was studied. The expression RANKL downstream signaling pathway including p38, JNK, ERK1/2 and IκB-α were detected by western blot. The results were shown in Fig. 5. The expression of phosphorylated p38 (p-p38) in the three experimental groups was lower than that in the RANKL (+) group, but the expression of p-JNK and p-ERK1/2 in the three experimental groups showed no obvious difference compared with the RANKL (+) group. In addition, the expression of IκB-α protein was up-regulated in Sr-SBG group and SrCl_2_ group compared with RANKL (+) group, indicating that both groups containing Sr element (Sr-SBG and SrCl_2_ group), could activate the expression of IκB-α and inhibit NF-κB signaling pathway. However, SBG group without Sr may inhibit osteoclast differentiation mainly through p38 signal pathway.

### Sr-SBG suppressed cell nuclei translocation of NFATc-1 in RANKL-induced RAW 264.7 cells

**Figure 6:**
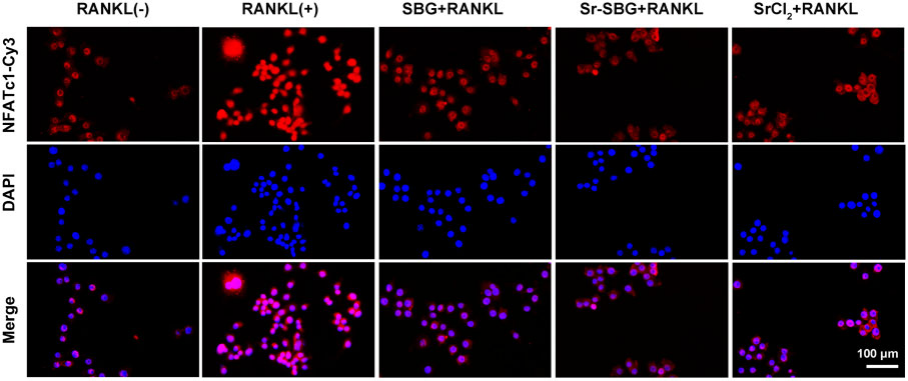
Location of NFATc1 identified by immunofluorescence assay of RAW264.7 cells cultured with SBG extract, Sr-SBG extract and SrCl_2_ supplemented with 50 ng/ml RANKL for 24 h.

NFATc1 is a key downstream signal molecule in RANKL-induced osteoclast differentiation. In the absence of RANKL stimulation, NFATc1 is mainly located in the cytoplasm. After being treated with RANKL, NFATc1 enters into the nucleus and activates the mRNA expression of osteoclastogenesis-related genes. It could be seen from Fig. 6. that a large number of NFATc1 entered into the nucleus in RANKL (+) group, while in SBG group, Sr-SBG group and SrCl_2_ group, translocation of NFATc1 into nucleus decreased, indicating that Sr-SBG, SBG and SrCl_2_ could all inhibit osteoclast differentiation to a certain extent.

### Discussion

Bioactive materials with unique biological active ions and special structures play important roles in repairing bone defects [6, 9]. As an extraordinary bioactive material for bone regeneration, BG was developed for potential bone and dental repair applications. In previous study, Sr-SBG has been found to significantly up-regulate osteogenesis-related genes expression [15, 16]. Nevertheless, the impact of Sr-SBG on osteoclast differentiation and the specific molecular mechanism is still unavailable. The aim of this study is to reveal the interaction between osteoclast precursor cells and Sr-SBG under clinical circumstance in the processes of bone repair. Therefore, as the structure and size of Sr-SBG and SBG are similar, materials extract of SBG and Sr-SBG, as well as Sr ion alone were prepared and applied to study the effect of materials on osteoclastogenesis. It is worth mentioning that the rate of apatite formation which is important for bone osseointegration has a negative correlation with Sr content of Sr-SBG, hence, 6% Sr-SBG was chosen for this study. To explore the ion extracts on the osteoclast differentiation, the ion concentrations in control group and three experimental groups (SBG, Sr-SBG and SrCl_2_) were tested. The major difference between BG-based groups (Sr-SBG and SBG) and other groups was that Si ion concentration from Sr-SBG group and SBG group were similar and significantly increased, whereas no Si ion existed in control group and SrCl_2_ group. In addition, the ion concentration of Sr in two Sr containing group (Sr-SBG and SrCl_2_) was identical, yet there was no Sr ion in the other two groups. Also, the Ca and P ion concentration in Sr-SBG and SBG group were significantly decreased, which might attribute to the formation of HCA layer on the surface of materials. Murine macrophage cell line RAW 264.7 was served as osteoclast precursor cell, which can differentiate into osteoclasts mediated by RANKL. This culture system was proved to be available and valuable for evaluating osteoclast differentiation in investigating the impacts of materials on osteoclastogenesis [36].

Our results showed that Sr-SBG, SBG and Sr ion alone could all suppress RANKL-induced osteoclastogenesis to some extent *in vitro*, with the declined number of TRAP-positive cells, and the formation of F-actin ring obstruction. Furthermore, Sr-SBG has been shown to have a stronger ability to inhibit osteoclast differentiation than SBG and SrCl_2_. Previous studies have shown that Sr ion can inhibit osteoclast differentiation by inhibiting NF-κB signaling pathway, and Si ion also plays an important role in inhibiting osteoclast differentiation. On the basis of this work, it can be suspected that the strontium-doped Sr-SBG may inhibit the differentiation of osteoclasts by NF-κB signal pathway and other signaling pathways induced by Si ions. Therefore, we studied the signal pathway downstream of RANKL after the binding of RANKL to RANK on macrophage surface. p38, ERK 1/2 and JNK are three important downstream pathways under RANKL-RANK signaling, which play important role in osteoclastogenesis [22, 23]. The present study showed that all of Sr-SBG, SBG and SrCl_2_ could inhibit the phosphorylation of p38, among which Sr-SBG had the greatest inhibitory effect on the phosphorylation of p38, but all of three experimental groups had no obvious inhibitory effect on the phosphorylation of JNK and ERK1/2 proteins. This may be due to the fact that both Sr ion and Si ion can inhibit the phosphorylation of p38, while both Sr ion and Si ion exist in Sr-SBG, thus they can play a synergistic role in inhibiting p38 phosphorylation. In addition, Sr-SBG and SrCl_2_ can also up-regulate the expression of IκB-α protein, IκB-α protein can regulate the activation of NF-κB. NF-κB can form complex with IκB-α when there are active molecules (such as IL1 β and TNF α) that can stimulate the activation of NF-κB in the environment. The complexes of NF-κB and IκB-α were depolymerized and transferred to the nucleus and the transcription of the target gene was initiated. Therefore, the detection of the significant degradation of IκB-α can verify whether NF-κB is activated. With the presence of Sr ions in Sr-SBG and SrCl_2_, the degradation of IκB-α is inhibited, thus the activation of NF-κB is inhibited.

**Figure 7:**
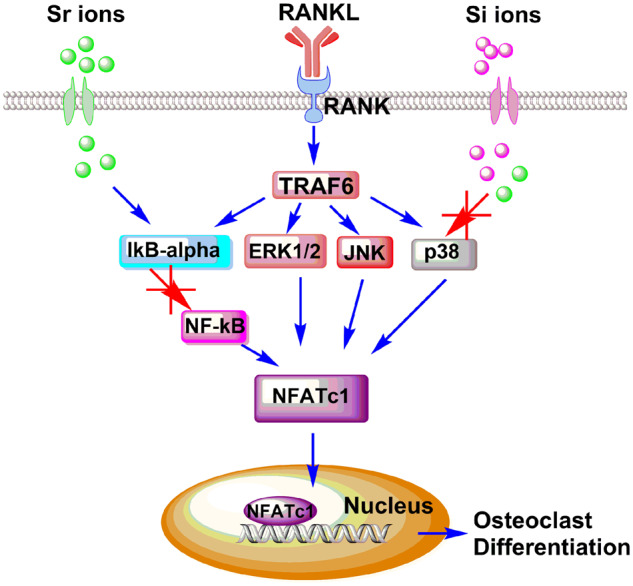
Proposed mechanism of inhibited RANKL-induced osteocalstogenic differentiation of Sr-SBG through blocking NF-κB signaling pathway and p38 signaling pathway which would result in decreased translocation of NFATc1 and eventually inhibit osteoclastogenesis.

On the RANKL-RANK signaling pathway and the downstream signaling pathways, mature and differentiation of RANKL-induced osteoclastogenesis is closely related associated with the mRNA expression level of transcription factors including NFATc1 [37]. According to our immunofluorescence staining results of NFATc1, all of three experimental groups could block the nuclear translocation of NFATc1 in RAW 264.7 cells, suggesting that both p38 pathway and NF-κB pathway suppress osteoclast differentiation through inhibiting the nuclear translocation of NFATc1. In conclusion, the molecular mechanism of Sr-SBG inhibition of osteoclast differentiation is the inhibition of NF-κB pathway through Sr ion activated IκB-α and the synergistic effect of Sr and Si ions on p38 pathway, which resulted in inhibited nuclear translocation of NFATc1 (Fig. 7).

## Conclusions

In summary, the inhibitory functions of Sr-SBG on RANKL-induced osteoclastogenesis from RAW 264.7 cells were investigated *in vitro*. Moreover, the underlying molecular mechanism of the suppression effect of Sr-SBG was also clarified. The results showed that SBG and Sr ion alone could inhibit osteoclastogenesis to certain extent, while Sr-SBG with combined effect of SBG extract and Sr ion showed the strongest inhibitory effect on osteoclast differentiation by disrupting the RANKL-activated p38 signaling and NF-κB signaling pathways. Our findings might offer possible mechanistic explanation for the effect of Sr-SBG on enhanced bone regeneration. Furthermore, it might provide a significant guidance for designing better bone repair materials.

## Supplementary Material

rbaa004_Supplementary_Data
