# Supplementary material for: Strontium-substituted sub-micron bioactive glasses inhibit ostoclastogenesis through suppression of RANKL-induced signaling pathway
Source: Regen Biomater. 2020 Mar 30;7(3):303–11. doi: 10.1093/rb/rbaa004 (PMC7266663; doi:10.1093/rb/rbaa004)
Supplement: rbaa004_Supplementary_Data [file rbaa004_supplementary_data.zip › rbaa004-Suppl_Data/Supplementary Files.docx]

Supplementary Materials for

**Strontium-substituted submicron bioactive glasses inhibit ostoclastogenesis through suppression of RANKL-induced signaling pathway**

Deqiu Huang ^2^, Fujian Zhao ^3^, Wendong Gao ^3^ Xiaofeng Chen ^3^, Zhouyi Guo ^2^, Wen Zhang ^1,*^

^1^Department of Medical Biotechnology, School of Basic Medical Sciences, Guangzhou University of Chinese Medicine, Guangzhou, Guangdong, P.R. China;

^2^MOE Key Laboratory of Laser Life Science & SATCM Third Grade Laboratory of Chinese Medicine and Photonics Technology, College of Biophotonics, South China Normal University, Guangzhou 510631, Guangdong, China;

^3^Department of Biomedical Engineering, School of Materials Science and Engineering, South China University of Technology, Guangzhou 510641, Guangdong, China

*Correspondence address. Department of Medical Biotechnology, School of Basic Medical Sciences, Guangzhou University of Chinese Medicine, Guangzhou 510006, Guangdong, P.R. China. E-mail: zwzhangwen@163.edu.cn (W. Z.)


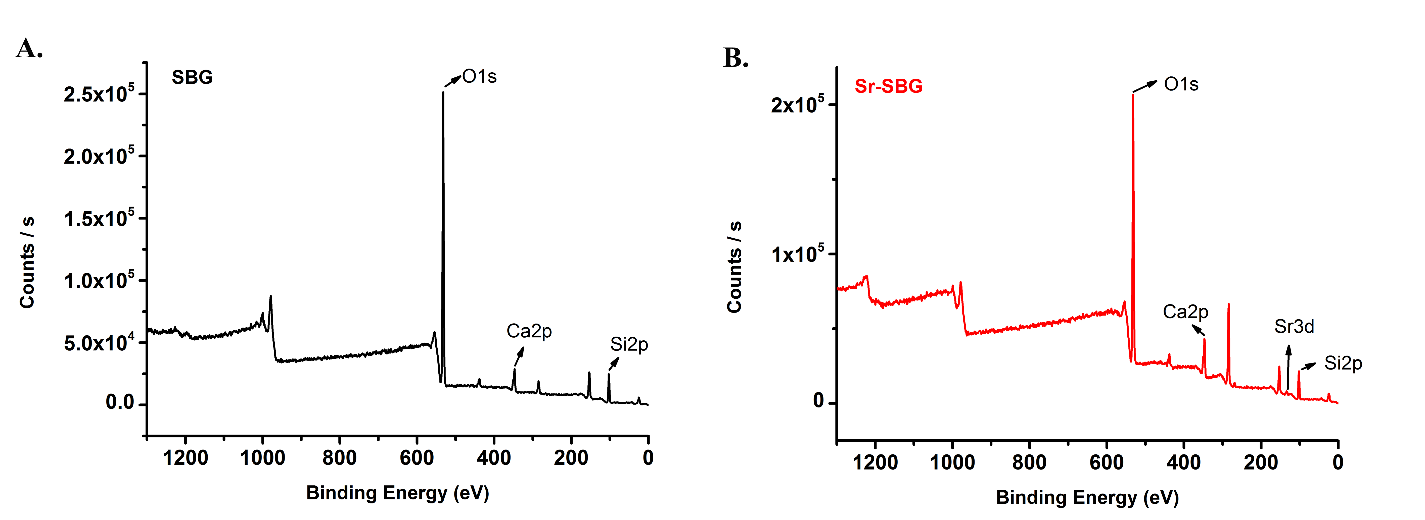


**Figure S1.** XPS characterization of SBG and Sr-SBG.

**

**

**Figure S2.** Cell proliferation of RAW 264.7 cells after 1, 3 and 5 days of culture with SBG extract, Sr-SBG extract and SrCl_2_. (*p < 0.05 vs control group)

**Table S1.** Average diameter and Zeta potential of SBG and Sr-SBG.

| **Sample** | **Average diameter (nm)** | **Zeta potential (mV)** |
| --- | --- | --- |
| SBG | 401.0 | -21.3 |
| Sr-SBG | 534.6 | -29.4 |
