# Supplementary material for: Strontium-substituted sub-micron bioactive glasses inhibit ostoclastogenesis through suppression of RANKL-induced signaling pathway
Source: Regen Biomater. 2020 Mar 30;7(3):303–11. doi: 10.1093/rb/rbaa004 (PMC7266663; doi:10.1093/rb/rbaa004)
Supplement: rbaa004_Supplementary_Data [file rbaa004_supplementary_data.zip › rbaa004-Suppl_Data/Detailed Response to Editors and Reviewers.docx]

Dear Dr. Nicholas Peppas and Reviewers,

Thank you very much for your letter and reviewers’ comments concerning our manuscript entitled “Strontium-substituted submicron bioactive glasses inhibit ostoclastogenesis through suppression of RANKL-induced signaling pathway” (RB-2019-078). These comments are very valuable and helpful for revising and improving our paper. We have studied these comments carefully and made corrections according to the comments. We hereby submit a revised version of the manuscript, and the modified portion are marked in red. We hope that the revision will meet with your approval. The main corrections in this manuscript and the responds to the reviewer’s comments are listed as follows.

***Responds to reviewer’s comments:***

**To Reviewer *1*:**

**Comments:**

*This paper presents the potential impact and underling mechanism of strontium-substituted submicron bioactive glass (Sr-SBG) on RANKL-induced osteoclast activation and differentiation in vitro. This is a carefully done study and the findings are of considerable interest. The paper is well arranged and thus it merits to be published. Just, I suggest some minor modifications before publication:*

*1. Why do the authors choose Sr-SBG with 6% Sr molar content for the research? It should be briefly clarified in the text.*

**Response:** Thank you for your positive comments. As for the referee’s concern, we have added a brief explanation in the *Results and Discussion section 3.8* about the reason why we choose 6% Sr-SBG in the revised manuscript which is marked in red and also listed as follows.

It is worth mentioning that the rate of apatite formation which is important for bone osseointegration has a negative correlation with Sr content of Sr-SBG, hence, 6% Sr-SBG was chosen for this study.

*2. In* *Results and Discussion section 3.5, it is necessary to add a description of the reason why the downregulation of NFATc1 and c-fos was earlier than that of TRAP and Cathepsin K.*

**Response:** Thank you for your comments. We have added *the reason why the downregulation of NFATc1 and c-fos was earlier than that of TRAP and Cathepsin K* in Results and Discussion section 3.5 in the revised manuscript which is marked in red and also listed as follows.

The earlier gene expression change of *NFATc1* and *c-fos* than *TRAP*, *Cathepsin K* and *MMP9* in osteoclastogenesis should be ascribed to the sequential events in osteoclast differentiation that *NFATc1* cooperates with other transcriptional partners to activate osteoclast-specific genes such as *TRAP*, *Cathepsin K* and *MMP9.*

*3. The introduction section should be expanded to include more references related to the downstream signaling pathways after RANKL binding to RANK.*

**Response:** Thank you for your suggestion. We have added some more related references about the downstream signaling pathways after RANKL binding to RANK in the revised manuscript and marked these in red and also listed as follows.

27.	Xing L, Schwarz EM, and Boyce BF. Osteoclast precursors, rankl/rank, and immunology*.* *Immunol Rev* 2005; **208:** 19-29.

28.	Tanaka S. Signaling axis in osteoclast biology and therapeutic targeting in the rankl/rank/opg system*.* *Am J Nephrol* 2007; **27:** 466-78.

29.	Leibbrandt A and Penninger JM. Rankl/rank as key factors for osteoclast development and bone loss in arthropathies*.* *Adv Exp Med Biol* 2009; **649:** 100-13.

30.	Ciacli C and Puschita M. Rankl/rank/opg molecular complex--control factors in bone remodeling in psoriatic arthritis*.* *Rev Med Chir Soc Med Nat Iasi* 2011; **115:** 354-60.

*4. There is at least one superscript and subscript spelling error in the manuscript, such as, in page 6, Line 50 and 52, “SiO2, CaO, and P2O5” would be “SiO2, CaO, and P2O5”; In page 8, Line 26, “ 5% CO2” would be “ 5% CO2” ; In page 8, Line 54, “3 × 103 cells/well” would be “3 × 103 cells/well”. Please check the manuscript carefully.*

**Response:** Thank you! The manuscript has been carefully checked and the spelling errors were corrected in the revised manuscript.

*5. On page 4, the last word osteoclasts would be deleted from this sentence “**Bone remodeling is regulated by osteogenesis and osteoclastogenesis through the continuous osteoblastic formation of new bone and osteoclastic resorption of old bone osteoclasts”.*

**Response:** Thank you for your kind suggestion. We have modified the sentence and deleted the last word osteoclasts in the revised manuscript. The sentence has been replaced by “*Bone remodeling is regulated by osteogenesis and osteoclastogenesis through the continuous osteoblastic formation of new bone and osteoclastic resorption of old bone.”*

Sincerely yours,

Dr. Wen Zhang

E-mail: zwzhangwen@163.com
